# Evaluation of 3D-Printed Thermoplastic Polyurethane Nerve Guidance Conduits

**DOI:** 10.3390/polym18151865

**Published:** 2026-07-29

**Authors:** Alexis B. Sabido-Barahona, Rossana F. Vargas-Coronado, Claudia Vásquez-López, Abraham J. Cisneros-Mejorado, Rainald Pablo Ordaz, Reinher Pimentel-Domínguez, Antònia Colom-Casasnovas, Rogelio O. Arellano, Juan V. Cauich-Rodríguez, Angel Marcos-Fernández

**Affiliations:** 1 Materials Department, Centro de Investigación Científica de Yucatán, A. C., Calle 43 No. 130 x 32 y 34, Col. Chuburná de Hidalgo, Mérida 97205, YUC, Mexico; alexis.sabido@estudiantes.cicy.mx (A.B.S.-B.); ross@cicy.mx (R.F.V.-C.); 2 Departamento de Física, Universidad de Sonora, Encinas y Rosales, s/n., Hermosillo 83000, SON, Mexico; claudia.vasquez@iq.uson.mx; 3 Laboratorio de Neurofisiología Celular, Instituto de Neurobiología, Universidad Nacional Autónoma de México (UNAM), Boulevard Juriquilla 3001, Querétaro 76230, QRO, Mexico; acisneros@secihti.mx (A.J.C.-M.); pablo.ordaz88@comunidad.unam.mx (R.P.O.); a.colomcasasnovas@gmail.com (A.C.-C.); arellano.ostoa@comunidad.unam.mx (R.O.A.); 4 Centro de Física Aplicada y Tecnología Avanzada, Universidad Nacional Autónoma de México (UNAM), Boulevard Juriquilla 3001, Querétaro 76230, QRO, Mexico; reinherp@fata.unam.mx; 5 Institute of Polymer Science and Technology (ICTP-CSIC), Juan de la Cierva, 3, 28006 Madrid, Spain

**Keywords:** nerve guidance conduit, 3D printing, thermoplastic polyurethanes, peripheral nerve regeneration, fused deposition modeling

## Abstract

Fused deposition modeling (FDM) is an emerging trend for producing nerve guidance conduits (NGCs). This technique allows distinct designs and dimensions to mimic peripheral nerve architecture and promote nerve regeneration. In this study, commercially available TPU 90A and 95A filaments were used for the fabrication of hollow simple wall (non-porous), grooved and gyroid multichannel conduits. The segmented polyurethanes were identified as PBA-MDI-BO-based polyurethanes by ^1^H NMR, FTIR and Raman spectroscopy. Thermal analyses, such as DSC and TGA, demonstrated that both TPUs possess sufficient thermal stability to be processed safely under the printing conditions employed. Tensile mechanical tests accounted for their differences in hard segment content in agreement with the Shore A hardness. The measured elastic modulus, in particular, was within a range that may be advantageous for peripheral nerve repair. The gyroid multichannel design showed enhanced resistance to radial compression and highly interconnected internal structure that supported primary neural cell viability and preserved cellular functionality. Therefore, polyurethane-based nerve guidance conduits can be manufactured by FDM, rendering high printability, favorable mechanical performance without compromising biocompatibility.

## 1. Introduction

Peripheral nerves injuries (PNIs) are common and represent a challenging surgical condition, with approximately 4.13 million cases worldwide [1]. Neurotmesis is the most severe type of nerve injury, involving a complete loss of nerve continuity [2]. In such cases, direct repair is performed by suturing the ends of the damaged nerve; if it is not feasible or fails, a nerve graft or tubulization technique using nerve guide conduits (NGCs) is required. The gold standard for repairing damaged nerves is the autograft; however, it has several limitations, including donor site morbidity, limited availability, and size mismatch [3]. The clinical use of NGCs provides an alternative to autografts for nerve repair of defects longer than 3 cm. NGCs are biocompatible tubular structures designed to protect the nerve from the physiological environment and guide the axon to promote nerve regeneration [4,5]. These conduits are fabricated from multiple biomaterials, including natural polymers (chitosan, collagen, silk fibroin) and synthetic polymers (polyglycolic acid, polyvinyl alcohol, poly (D, L-lactide-co-ε-caprolactone)), as well as biologically derived materials such as extracellular matrix (ECM) from cadaveric nerve or porcine tissues [6,7]. Depending on the fabrication method and material, the design and architecture of NGCs can vary significantly, and therefore, so can their performance. NGCs can be classified into five main designs: hollow simple wall (non-porous), porous, grooved and multichannel [8,9]. All these designs influence nerve regeneration, and each presents specific advantages and limitations. Conventional fabrication methods of NGCs include dip-coating, solvent casting, freeze-drying, electrospinning, particle leaching, phase separation, self-assembly, and micropatterning. For instance, solvent casting and dip-coating typically produces dense tubes, whereas freeze-drying, electrospinning, phase separation and particle leaching generate porosity within the tube wall. Currently, research focuses on modifying and optimizing NGCs’ structure by combining materials, novel morphologies and advanced fabrication techniques to improve regenerative outcomes.

3D printing or additive manufacture (AM) is a process that manufactures structures using layer-by-layer deposition. This technique is a promising approach to overcome the limited control over scaffold architecture observed in tissue engineering using conventional fabrication methods. 3D printing provides numerous advantages such as customized scaffolds with less wasted material, freedom of geometry, high reproducibility and the capability to generate complex architectures that closely mimic native tissues [10,11]. In general, NGCs are fabricated using three principal AM approaches: photopolymerization (stereolithography, digital light processing), material extrusion (fused deposition modeling, extrusion bioprinting) and material jetting (inkjet bioprinting, electrohydrodynamic jet, laser bioprinting). Fused deposition modeling (FDM) involves parameters which must be optimized to achieve specific high-resolution structures with defined geometries: material extrusion temperature, bed temperature, nozzle size and printing velocity [12]. The choice of biomaterials is an important criterion that affects NGC properties, including mechanical performance, chemical resistance, printability and sterilization stability [10].

Polyurethanes (PUs) are versatile macromolecules that exhibit low cytotoxicity, biodegradability, and mechanical properties comparable to native tissues [13]. Thermoplastic polyurethanes (TPUs) can be processed into filaments suitable for FDM and consist of alternating hard and soft segments that can undergo phase separation, leading to the formation of microdomains that function as physical cross-links. Soft segments are typically composed of polyesters or polyethers, whereas hard segments result from the reaction between diisocyanates and short molecular chain extenders. The chemical structure and the morphology of the phase-separated structure determine properties such as flexibility, hardness, and mechanical strength.

The use of TPU-based NGCs fabricated via FDM is an emerging trend for producing tubular structures with complex designs and channels that mimic nerve architectures, demonstrating favorable cellular responses and promoting nerve regeneration in vivo with outcomes comparable to autograft. 3D-printed TPU can be used as a framework, which can be coated with a porous biomaterial to obtain a mechanically stable and porous nerve conduit. Alternatively, NGCs can be fabricated entirely from TPU using 3D models anatomically equivalent to native nerves that mimic the multichannel structure to achieve the desired structure and size.

In this study, commercial TPU filaments with different hardness values (95A and 90A) were chemically and biologically characterized to assess their suitability for nerve regeneration applications. Then, NGCs were fabricated and mechanically characterized according to the principal structural designs (hollow non-porous wall, grooved and multichannel). It has been reported that multichannel conduits show greater efficacy compared with hollow design. In particular, multichannel NGCs were fabricated using a gyroid infill pattern. Gyroid architecture is based on an interconnected porous structure found in certain biological membranes and has been extensively investigated in tissue engineering scaffolds due to its high porosity, interconnected network, and favorable mechanical properties [14,15]. These characteristics have demonstrated promising potential for tissue regeneration. Furthermore, a gyroid inner structure may facilitate nutrient and oxygen diffusion while promoting the removal of metabolic waste. Its continuous and smooth surface may also support Schwann cell adhesion and migration, as well as endothelial cell infiltration and neovascularization. To the best of our knowledge, the use of multichannel gyroid architectures in FDM-fabricated TPU nerve guidance conduits remains scarcely explored. Finally, infill density varied 10%, 20%, and 30% to evaluate the influence of gyroid infill density on the mechanical performance of conduits.

## 2. Materials and Methods

### 2.1. Materials

Commercial thermoplastic polyurethane (TPU) filaments (1.75 mm nominal diameter) were employed for 3D-printed NGCs. TPU 90A (PolyFlex_90A, Polymaker, Shanghai, China) [16] and TPU 95A (Ultimaker, Utrecht, the Netherlands) were used as received [17]. Specifically, TPU 95A was selected as the reference, as it has been previously investigated for tissue engineering applications, demonstrating favorable biocompatibility with fibroblasts, neurons, and Schwann cells, as well as suitable printability using FDM technology [18,19]. A second TPU 90A with lower Shore hardness was selected, and this allowed the evaluation of how variations in TPU composition affect the mechanical stability and flexibility of conduits, which are critical parameters for materials intended for peripheral nerve regeneration. Both filaments were selected from the same monomer composition (with different ratios) and processed under identical FDM conditions to minimize variations associated with manufacturing and processing parameters.

### 2.2. Chemical Characterization of TPU

The chemical composition of the TPUs was determined by proton nuclear magnetic resonance (^1^H-NMR) spectroscopy. Spectra were acquired on a Unity Plus 400 instruments (Varian Inc, Palo Alto, CA, USA) at room temperature, dissolving 10 mg of the filament in 1 mL of deuterated dimethyl sulfoxide (DMSO-d6) with gentle heating. All spectra were referenced to the residual solvent peak at 2.50 ppm. Complementarily, Fourier-transform infrared (FTIR) and Raman spectroscopy were conducted to further confirm the chemical structure and identify the functional groups. FTIR was performed using a Thermo Scientific Nicolet 8700 (Mountain, Waltham, MA, USA) spectrometer in attenuated total reflectance (ATR) mode (range of 4000–650 cm^−1^, resolution of 0.482 cm^−1^, and an average of 100 scans). Raman spectra were obtained using a Renishaw inVia Reflex spectrometer (Gloucestershire, UK) over a range of 4000–200 cm^−1^.

### 2.3. Thermal Characterization of TPU Filaments

Safe extrusion temperatures of TPU filaments were determined by thermogravimetric analysis (TGA) using a Perkin Elmer TGA-8000 (Drachten, The Netherlands). Measurements we performed from 50 °C to 650 °C, at a heating rate of 10 °C/min, under a nitrogen atmosphere. Differential scanning calorimetry (DSC-250 from TA Instruments, New Castle, DE, USA) was used to determine the glass transition (Tg) and melting temperature (Tm) of filaments and evaluate the phase separation. For this, approximately 10 mg of the sample was heated from −90 °C to 200 °C at a heating rate of 10 °C/min in a nitrogen atmosphere.

### 2.4. Accelerated Degradation of TPU

Accelerated degradation studies were conducted to assess the stability and chemical changes of TPU filaments. This study was conducted by immersing TPU in four different aqueous media (distilled water, 30% $H_{2}O_{2},$ acid medium (2M HCl), and alkaline medium (5N NaOH)) under reflux at 100 °C for 8 h. At the end of the test time, the remaining mass ($m_{r})$ was recovered, dried at room temperature and finally weighed. The percentage of degraded mass was determined based on the initial mass ($m_{i}$) according to Equation (1). Chemical changes after the degradation process were evaluated by obtaining FTIR spectra of degraded samples, following the methodology described in the chemical characterization section.

(1) $$\mathrm{Degraded}\;\mathrm{mass}\;\left[\%\right]=\frac{m_{i}-m_{r}}{m_{i}}\;\times \;100$$

### 2.5. Uniaxial Tension Test of Printed TPU

The tensile properties of the 3D-printed TPU were evaluated to better represent the material behavior after processing, including thermal exposure and extrusion during conduit fabrication. The layer orientation of specimens was aligned with loading direction. dog-bone-shaped specimens were printed in accordance with ASTM D638 [20] (Type IV), at 3:1 scale, using an extrusion temperature of 225 °C and a bed temperature of 35 °C. Samples were mounted on grips of a AGS-X Universal Testing Machine (Shimadzu,Tokyo, Japan) with a 100 N load cell at a crosshead speed of 100 mm/min. At least 10 of the printed specimens were tested. The evaluated mechanical properties included elastic modulus (E), maximum strength ($\sigma_{\max}$) and maximum deformation ($\epsilon_{\max}$). E was calculated from the slope of the linear region of the stress–strain curve between 1% and 3% strain.

### 2.6. 3D Printing of Nerve Guide Conduit with Different Morphologies

NGCs were printing using a Creality Ender-3 S1 Pro printer with TPU filaments of 90A and 95A hardness. Human-scale conduits were fabricated following the main structural designs reported in the literature [8,9,21], including hollow non-porous wall, hollow grooved (unidirectional grooves) and multichannel. The hollow and grooved conduit designs were created in AutoCAD 2026 (Autodesk Inc., San Rafael, CA, USA) and exported as STL. The STL files were imported into Creality Slicer 4.8.2 (Creality. Shenzhen, China), where printing parameters were defined. The dimension of the hollow conduit for the human model was as follows: outer diameter ${(d}_{e})$ of 8 mm, length (L) of 50 mm, and wall thickness (t) of 0.35 mm. In addition, a “spiralized” mode was used, consisting of a continuous helical (spiral) material deposition. For the grooved and multichannel conduits, the same length and outer diameter values previously mentioned were maintained, and a conventional layer-by-layer deposition mode was used. In particular, multichannel NGCs were produced using the STL file of the hollow conduit, and the gyroid infill pattern was implemented using the slicing software during the print preparation stage. All NGCs were printed in vertical orientation, with longitudinal axis aligned with the Z-axis of the printed NGC. A brim (1 mm) was generated during slicing to prevent displacement of conduit during printing and was removed after fabrication. Printing parameters are listed in Table 1.

### 2.7. Lateral Compression Test of 3D-Printed NGC

The lateral compression test was performed to evaluate the mechanical behavior of the printed conduits under implantation conditions. This test has been proposed to simulate the loads exerted by surrounding tissues. The test was conducted in quintuplicate (n=5) using an AGS-X Universal Testing Machine (Shimadzu, Kyoto, Japan) equipped with two parallel circular plates, a crosshead speed of 1 mm/s, and a 100 N load cell. The conduit was positioned such that the load was applied perpendicular to the longitudinal axis of the conduit until 50% deformation was reached. The specimens were held under compression for 60 s, and the outer diameter under compression ($d_{c}$) was subsequently measured. During compression test, load $F_{c}$ and displacement $\delta_{c}$ were recorded.

Currently, there is a proposed parameter to characterize the mechanical response of hollow NGCs: the structural compression modulus ($E_{s}$). Notably, both parameters were evaluated exclusively in hollow 3D-printed conduits. First, the diametral deformation was calculated according to Equation (2):

(2) $$\varepsilon_{d}=\frac{\delta_{c}}{d_{c}}$$

Subsequently, the force per unit length ($F_{e}$) (Equation (3)) was calculated and used as a structural parameter in lateral compression:

(3) $$F_{e}\;[N/\mathrm{mm}]\;=\frac{F_{c}}{L}$$

Using the values of $F_{e}$ and $\varepsilon_{d}$, the curves $F_{e}$ vs. $\varepsilon_{d}$, were generated, and the structural compression modulus $E_{s}$ [N/mm] was calculated from the slope under the linear region at low deformations (2.5–5%).

### 2.8. Indirect Viability Assay

As preliminary biological assessment, an indirect viability assay (extract method) was performed using human fibroblast CCL-116 Detroit. Culture medium was prepared using DMEM with low glucose content (Caisson Laboratories, Smithfield, UT, USA), 10% FBS (Biowest, Bradenton, FL, USA) and 1% streptomycin/penicillin (Sigma-Aldrich, St. Louis, MO, USA). Discs measuring 6 mm in diameter and 0.5 mm in height were designed and printed with TPU 90A and TPU 95A under the same printing parameters employed for the fabrication of the conduits. Material extracts were prepared by placing sterilized 3D-printed TPU (UV irradiation for 15 min on each side, and the procedure was repeated twice) at a concentration of 5 mg/mL in culture medium. Fibroblast were seeded in 96-well plates at a density of 10,000 cells per well in 100 μL of medium in an incubator (37 °C, 5% CO_2_) for 24 h. After this time, culture medium was replaced with 100 μL of extract, followed by incubation for 24, 36 and 72 h. At each time, 50 μL of the resazurin solution was added to cell culture and incubated for additional 6 h. Finally, resazurin reduction was measured spectrophotometrically at 570 and 600 nm with a Cytation 3 plate reader (Biotek). Cell viability percentage (Equation (4)) corresponded to the average of five independent wells (*n* = 5):

(4) $$\mathrm{Cell}\;\mathrm{Viability}\;\left[\%\right]=\frac{\left(C_{2}\times A_{1}\right)-(C_{1}\times A_{2})}{\left(C_{2}\times P_{1}\right)-(C_{1}\times P_{2})}\times 100$$

$C_{1}$ is the extinction coefficient of oxidized resazurin at 600 nm.

$C_{2}$ is the extinction coefficient of oxidized resazurin at 600 nm.

$A_{1}$ is the absorbance of the well measured at 570 nm.

$A_{2}$ is the absorbance of the well measured at 600 nm.

$P_{1}$ is the absorbance of the positive control measured at 570 nm.

$P_{2}$ is the absorbance of the positive control measured at 600 nm.

The reported values of $C_{1}$ y $C_{2}$ are 80,586 and 117,216, respectively.

The methodology for resazurin assay and the supplier specifications were based on reference [22]. A one-way analysis of variance (ANOVA) followed by Tukey’s post hoc test was performed to compare the mean cell viability among the different TPU filaments using Origin Pro 2018 software.

### 2.9. Direct Viability Assay and Cell Adhesion

Fibroblasts were cultured on printed TPU discs, and their viability was measured after 24 and 48 h using the spectrophotometric methodology described in the indirect viability assay (*n* = 5). Cell adhesion was evaluated qualitatively after using optical microscopy (OM) and scanning electron microscopy (SEM) imaging. Samples were collected at predetermined time points, washed with phosphate buffer and transferred to a 48-well plate. Each sample was incubated for 60 min at 4 °C and fixed with 300 µL of 4% glutaraldehyde (GTA) solution prepared from a 25% GTA reagent. Additionally, ethanol solutions at 50%, 60%, 70%, 80%, and 90% were prepared using absolute ethanol. After fixation, samples were washed again with phosphate buffer and subjected to a gradual dehydration series with ethanol, starting from 50% up to absolute ethanol, with each gradient lasting 15 min. During this process, care was taken to prevent sample drying, and ethanol changes were performed carefully along the well walls to preserve cell integrity. Finally, samples were immersed in isopropyl alcohol for 15 min and then left in the wells, submerged in isopropyl alcohol, inside an oven without temperature to allow for slow evaporation of the isopropyl alcohol to minimize structural disruption and shrinkage.

### 2.10. Primary Cortical Mixed Glial Cell Cultures

Primary mixed cortical glial cell cultures were prepared from neonatal Sprague Dawley rats (P0–P2) like previous reports [23]. These cultures were selected instead of immortalized cell lines because they provide a more physiologically relevant neural cellular environment for evaluating biomaterial–cell interactions. All procedures were approved by the Ethics Committee of the Instituto de Neurobiología (UNAM) and complied with NIH guidelines. Cortices were dissected in HBSS (Hank’s balanced salt solution; without calcium and magnesium) medium. The recovered tissue was disaggregated, first enzymatically by applying Trypsin (2.5%) and DNase (0.4%), and then mechanically using a 1 mL sterile syringe and 21G and 23G needles. Cells were seeded at 1–2 × 10^5^ cells per substrate onto UV-sterilized polymeric films or glass coverslips (control), previously coated with poly D-lysine (10 µg/mL). Cultures were maintained at 37 °C and 5% CO_2_ in IMDM (Iscove′s Modified Dulbecco′s Medium) medium supplemented with 10% fetal bovine serum. Medium was replaced every 48 h. Experiments were performed at 3 or 12 days in vitro (DIV).

### 2.11. Fluorescence Staining

Nuclear and cytoplasmic staining was performed at the corresponding days in vitro (DIV), and cultures were fixed with 4% paraformaldehyde for 20 min and washed with PBS. Nuclei were stained with DAPI (4 µg/mL). Cytoplasmic morphology was assessed using Sulforhodamine B (SRB; Sigma-Aldrich, St. Louis, MO, USA). Samples were mounted in Mowiol and imaged. Images were acquired using a Zeiss LSM880 confocal microscope equipped with 405 nm and 543 nm lasers.

### 2.12. Microscopy and Image Analysis of Glial Cell Cultures

Cells were imaged using confocal fluorescence microscopy, whereas polymeric substrates were characterized by confocal reflectance microscopy. Image acquisition was performed using an inverted laser scanning confocal microscope (Zeiss Axio Observer) equipped with an LSM 880 confocal module (Carl Zeiss, White Plains, NY, USA). Fluorescence imaging was performed using acquisition parameters optimized for DAPI and SRB, employing 405 and 543 nm excitation lasers and detection windows of 410–515 nm and 585–730 nm, respectively. Reflectance imaging was acquired using a 633 nm excitation laser with a detection range of 630–636 nm and a partial beam splitter, following the previously reported SCORE method [24]. Z-stack images were acquired using a 20× objective with a step size ranging from 0.4 to 1.5 µm. Maximum-intensity projections and three-dimensional (3D) reconstructions were generated using ZEN Blue (Zeiss, White Plains, NY, USA) and ImageJ/Fiji software, NIH (v1.54p, http://imagej.org). Cell density was quantified by automated counting of DAPI-positive nuclei in ImageJ. For each experimental condition, at least three substrates from three independent cultures were analyzed. Data are expressed as mean ± SD. Statistical comparisons were performed using one-way ANOVA followed by Tukey’s post hoc test. Differences were considered significant at *p* < 0.05. Analyses were performed using GraphPad Prism 10.

## 3. Results and Discussion

### 3.1. Chemical Characterization

**Figure 1:**
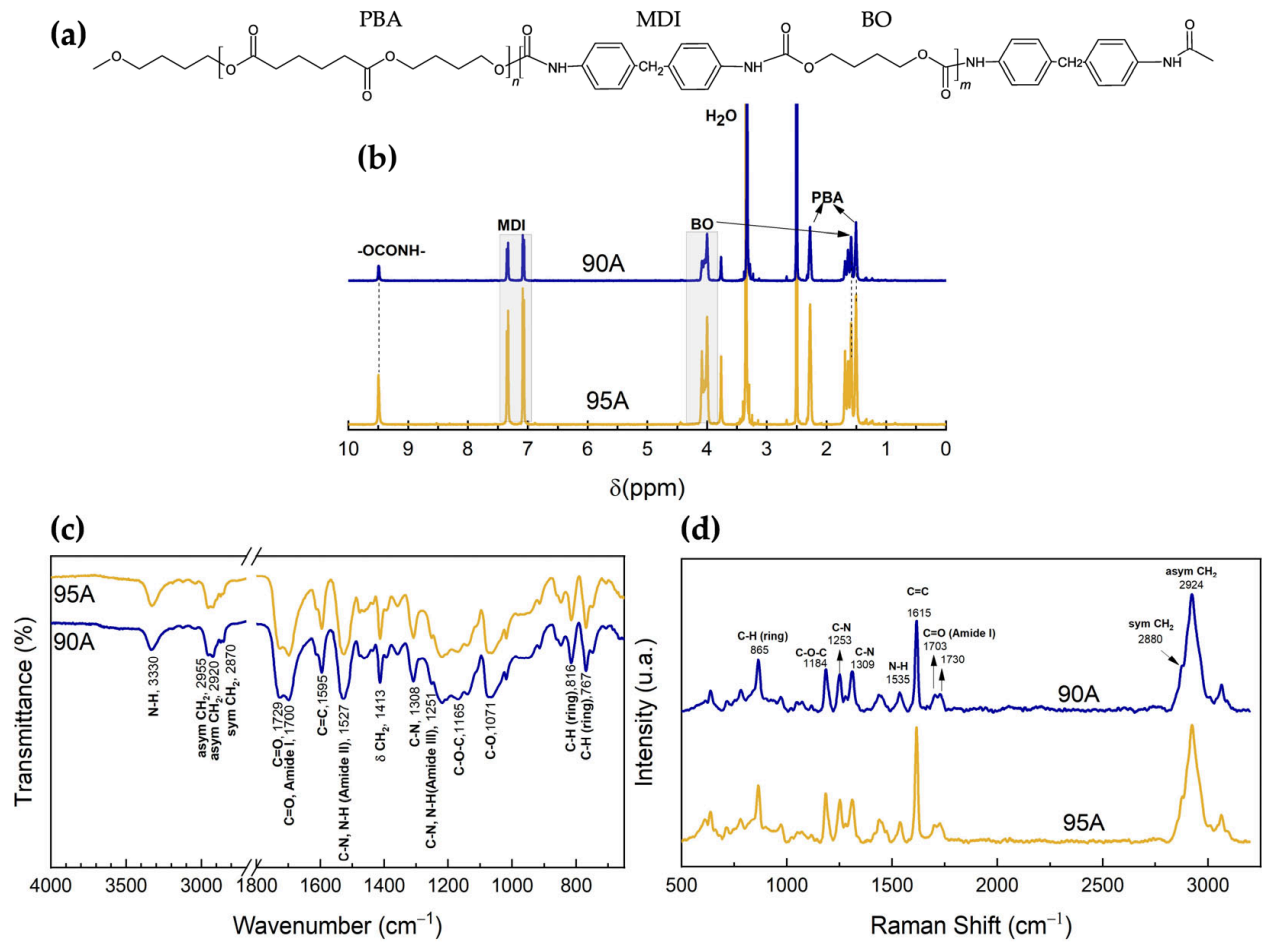
(**a**) Chemical structure of TPU filaments, (**b**) ^1^H-NMR, (**c**) FTIR and (**d**) Raman spectra of TPU filaments.

The ^1^H NMR spectra (Figure 1b) provided information about the chemical composition of the monomers, including the type of macroglyol, diisocyanate and chain extender used in the TPU filaments with varying Shore A hardness. First, NH protons of urethane group correspond to the signal at 9.49 ppm. For both filaments, the identified macroglyol was poly (butylene adipate) (PBA) with signals at 2.28 and 1.51 ppm for adipate [25]. In addition, the aromatic peak signals at 7.35, 7.33, 7.08 and 7.06 ppm [26], along with the aliphatic peak at 3.80 ppm, correspond to 4,4′-methylene bis (phenyl isocyanate) (MDI). Finally, for 1,4-butanediol (BO), the signals at 4.09 and 1.69 ppm correspond to its reaction with MDI, resulting in urethane linkages at both ends. Additionally, the peaks at 4.01, 1.65 and 1.59 ppm correspond to its reaction with adipate molecules (BO-adipate) and, therefore, to ester groups at both ends [26,27]. Although PolyFlex 90A and Ultimaker TPU 95A have the same chemical composition, PBA, MDI and BO (see the chemical structure in Figure 1a), have different Shore A hardnesses. This was justified by the differences in hard and soft segments. The percentage of rigid segment content (HS) was estimated from the molar ratio of MDI and BO to MDI, BDO and PBA by analyzing the proton integral values (PIVs) following Equation (5):

(5) $$\mathrm{HS}\;[\%]\;=\frac{\mathrm{PIV}\;\mathrm{of}\;\mathrm{MDI}\;+\;\mathrm{PIV}\;\mathrm{of}\;\mathrm{BO}}{\mathrm{PIV}\;\mathrm{of}\;\mathrm{Total}\;\mathrm{TPU}}$$

The calculated hard segment contents were approximately 45.5% and 54.1% for the 90A and 95A TPU filaments, respectively. These values are consistent with the hardness classification provided by the manufactures, where a higher hard segment content corresponds to a higher Shore A hardness. It is generally accepted that the hard and soft segment content influences phase separation and mechanical properties and, in consequence, the performance of 3D-printed structures [28].

In Figure 1c, FTIR analysis revealed the characteristic polyurethane vibrations in both filaments at 3330 ${\mathrm{cm}}^{-1}$ (N-H stretching), 1700 ${\mathrm{cm}}^{-1}$ (C=O, Amide I), 1527 ${\mathrm{cm}}^{-1}$ (C-N, N-H, Amide II), 1308 ${\mathrm{cm}}^{-1}$ (C-N), and 1251 ${\mathrm{cm}}^{-1}$ (C-N, Amide III) [29]. In addition, the signals corresponding to the aliphatic chain of PBA were observed at 2955/2920 ${\mathrm{cm}}^{-1}$ (asymmetric ${\mathrm{CH}}_{2}$), 2870 ${\mathrm{cm}}^{-1}$ (symmetric ${\mathrm{CH}}_{2}$), 1729 ${\mathrm{cm}}^{-1}$ (C=O free ester), 1165 ${\mathrm{cm}}^{-1}$ (C-O-C), and 1071 ${\mathrm{cm}}^{-1}$ (C-O). Finally, the characteristic bands of MDI appeared at 1595 ${\mathrm{cm}}^{-1}$ (C=C), 1413 ${\mathrm{cm}}^{-1}$ (aromatic *δ* (C-H) bending), and 816 ${\mathrm{cm}}^{-1}$ y 767 ${\mathrm{cm}}^{-1}$ (out-of-plane aromatic C-H bending vibrations) [30]. The bonds and functional groups identified by FTIR were also confirmed by Raman spectroscopy (Figure 1d), where the following signals were observed for both TPU filaments: 865 ${\mathrm{cm}}^{-1}$ (ring C-H), 1184 ${\mathrm{cm}}^{-1}$ (C-O-C), 1253 ${\mathrm{cm}}^{-1}$ and 1309 ${\mathrm{cm}}^{-1}$ (C-N), 1535 ${\mathrm{cm}}^{-1}$ (C-N, N-H), 1615 ${\mathrm{cm}}^{-1}$ (C=C), 1703 ${\mathrm{cm}}^{-1}$ y 1730 ${\mathrm{cm}}^{-1}$ (free and hydrogen-bonded amide I C=O,), 2880 ${\mathrm{cm}}^{-1}$ y 2955/2924 ${\mathrm{cm}}^{-1}$ (symmetric and asymmetric stretching ${\mathrm{CH}}_{2}$).

### 3.2. Thermal Characterization

**Figure 2:**
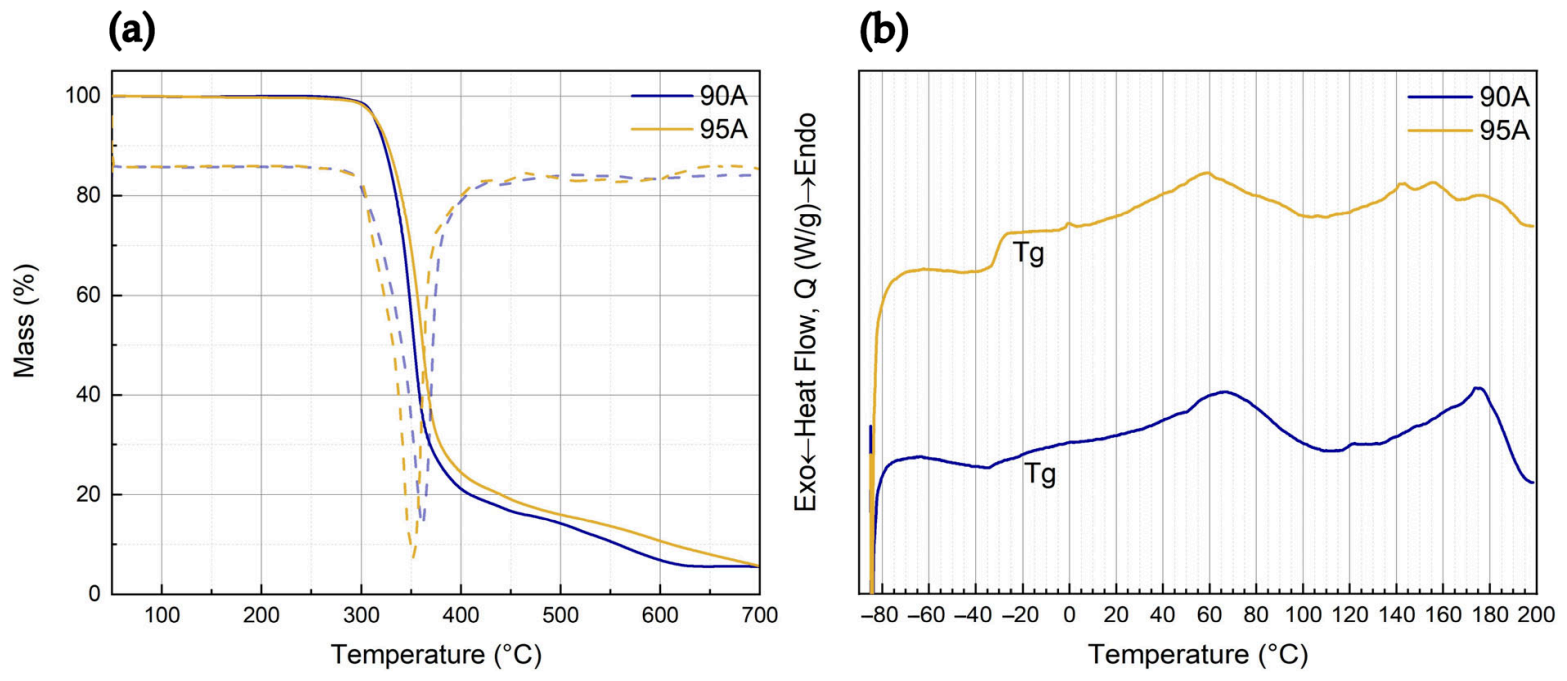
(**a**) TGA and (**b**) DSC thermograms of TPU filaments.

The TGA thermograms (Figure 2a) showed a single step for the thermal degradation profile for both TPUs, without a difference in the degradation of hard and soft segments. The temperature for the start of degradation is a critical parameter for defining safe extrusion conditions in 3D printing, as the material is expected to remain chemically stable below this temperature. TPU filaments started to degrade at approximately 290 °C, while the temperatures of maximum degradation rate were 352 °C for TPU 90 and 361 °C for TPU 95A. Furthermore, the nozzle temperature selected for NGC fabrication (225 °C) was well below the onset of thermal degradation; therefore, the extrusion process is not expected to induce thermal degradation of the material.

On the other hand, in the DSC thermograms of TPU filaments (Figure 2b), it was observed that Tg decreased from −30 °C (95A) to −35 °C (90A) when hard segment content decreased. Interestingly, both TPU filaments exhibited a broad endothermic transition extending from approximately 20 °C to 100 °C, with a maximum centered near 60 °C. Comparison with the DSC thermogram of pure PBA (Figure S1, Supplementary Material) suggests that the endothermic event up to approximately 60 °C is mainly associated with the melting of crystalline PBA domains. The higher-temperature region of the endotherm transition may also include contributions from thermal events related to hard segments. Therefore, although the peak maximum at approximately 60 °C is attributed to PBA melting, the broad endothermic region is likely the result of overlapping transitions arising from both soft and hard segment domains.

Additionally, a well-defined melting temperature Tm was not observed in TPU samples. Instead, multiple endothermic transitions appeared above 120 °C, which may be associated with different sizes of hard segment crystals, melting–recrystallization processes occurring during heating or the mixing of soft and hard segments [31]. This behavior has previously been reported for TPUs based on the same hard segment chemistry (MDI─BO) [32].

### 3.3. Accelerated Degradation Studies

**Figure 3:**
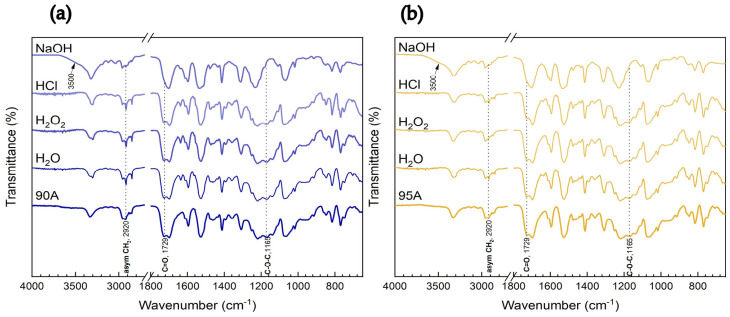
FTIR spectra of (**a**) TPU 90 A and (**b**) 95A degraded in different media.

Hydrolytic degradation was conducted at a temperature higher than the physiological condition to accelerate degradation while preserving the degradation mechanism of the TPU. The weight change of printed TPUs (90A and 95A) after exposure to $H_{2}O$, $H_{2}O_{2}$ and HCl was negligible (<1%). FTIR spectra in Figure 3 revealed a change on the 2920 ${\mathrm{cm}}^{-1}$ (asymmetric ${\mathrm{CH}}_{2}$) absorption, probably associated with the degradation of short BO or PBA chain segments. However, no other significant changes in the chemical composition of TPU were observed. These findings may be attributed to the relatively short duration of the degradation study (24 h under reflux), and longer exposure times may be required to detect measurable chemical modifications. On the other hand, both TPUs were hydrolytically degraded in NaOH medium. Under these conditions, the bands at 1729 ${\mathrm{cm}}^{-1}$ (C=O free ester) and 1165 ${\mathrm{cm}}^{-1}$ (C-O-C) from PBA disappeared, which is consistent with the observed mass loss of 71.0 ± 0.1 for TPU 90A and 33.0 ± 0.7 for TPU 95A. Additionally, the appearance of a broad band around 3500 ${\mathrm{cm}}^{-1}$ confirms the formation of hydroxyl groups resulting from the hydrolysis of the polyester soft segment. Hydroxide anions in the NaOH medium acted as strong nucleophiles, attacking the ester groups from PBA soft segments and promoting alkaline hydrolysis [33], although higher hard segment content reduced permeability and diffusion due to a more compact microphase separated structure [34]. Since TPU 90A contains a higher proportion of PBA and a lower proportion of hard segments than TPU 95A, it exhibits a greater number of hydrolysable ester bonds and increased accessibility to hydroxide ions, thereby promoting degradation. Short-term degradation assays with other commercial polyester-based TPUs showed similar results in alkaline medium [35].

### 3.4. Uniaxial Tension Analysis

**Figure 4:**
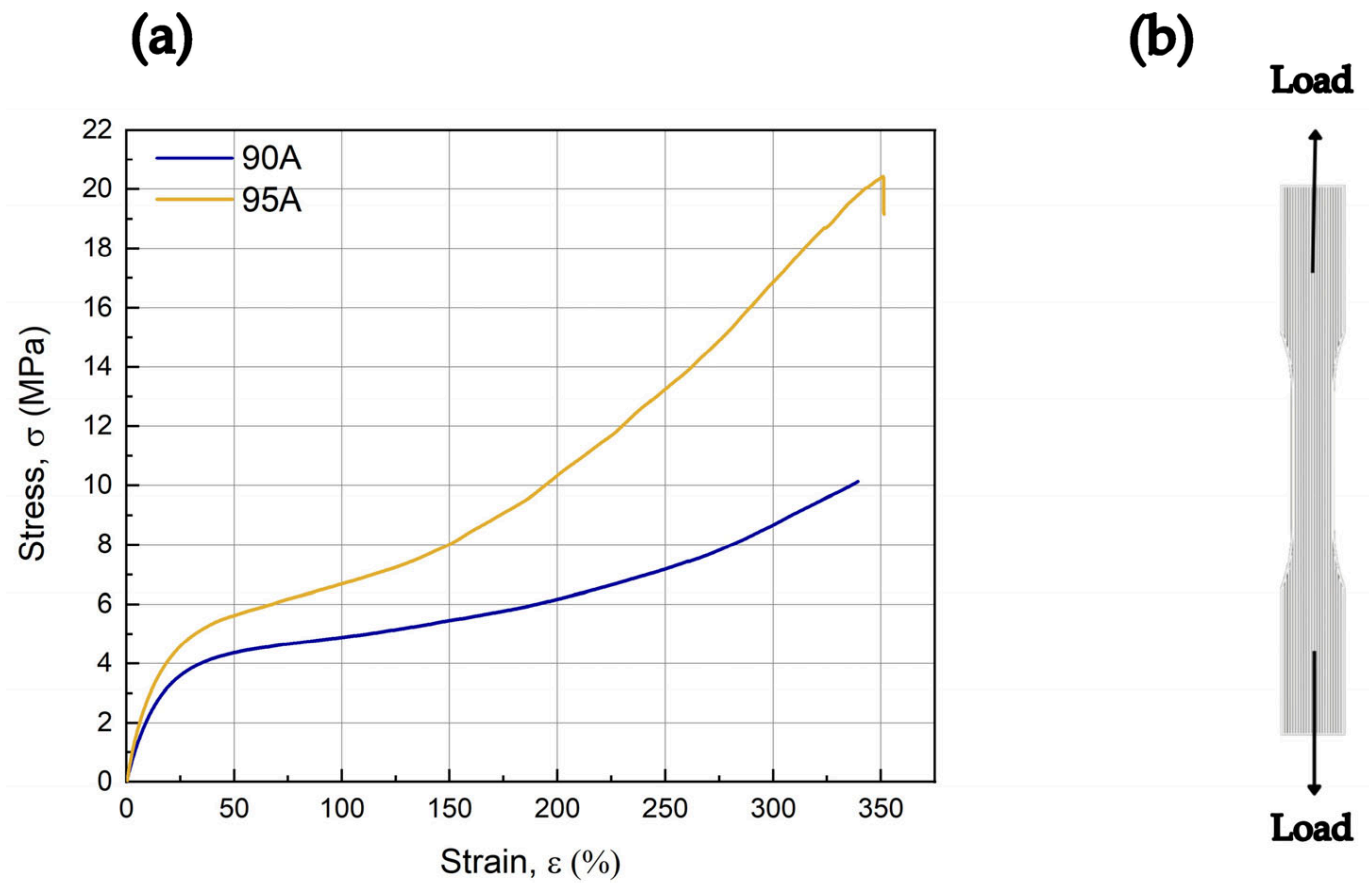
Uniaxial test. (**a**) Stress–strain curves of TPUs and (**b**) 3D-printed dog-bone-shaped specimens.

Figure 4 shows typical stress–strain curves for each TPU (90A and 95A). Uniaxial tension test of printed TPU dog bone specimens revealed that the mechanical properties increase with the increase in Shore A hardness. Given that TPU filaments 90 A and 95 A possess the same chemical constituents but different monomer molar ratios, the observed differences in elastic modulus, mechanical strength and maximum strain (See Table 2) can be attributed to variation in the hard/soft segment ratio. This trend is consistent with the higher hard segment content of the 95A TPU, resulting in a greater proportion of carbamate groups, which promotes stronger intermolecular interactions and improves the mechanical properties [36]. Consequently, the 95 A TPU exhibited superior mechanical performance compared with the 90A TPU.

Since the tensile specimens and NGCs were fabricated from the same TPU filament under identical printing conditions, the tensile properties are expected to reflect the intrinsic mechanical behavior of the printed material. Therefore, they provide a relevant indication of the mechanical environment and are considered representative of the substrate stiffness experienced by cells, although the overall mechanical response of the conduits may also be influenced by their geometry. Ideally, NGC must be manufactured using materials with mechanical properties that mimic the peripheral nerves. The printed TPUs’ elastic moduli (20.8 ± 1.4 MPa for 90A and 30.6 ± 1.7 MPa for 95A) are above the range reported for peripheral nerves (8–6 MPa) [8], but this can be reduced considering their long-term degradation during in vivo implantation. In this regard, elastic modulus is a key parameter influencing cell behavior and nerve regeneration, as substrate stiffness has been shown to affect cell adhesion, proliferation, differentiation and axonal growth [37]. In general, stiff materials do not promote good adhesion, but this was not the case as demonstrated later. In terms of mechanical strength, the printed TPUs exhibited tensile strength values (10.1 ± 0.1 MPa for 90A and 22.7 ± 1.5 MPa for 95A) which are higher than those reported for native peripheral nerves (6.78 ± 0.5–8.54 ± 3.30 MPa) [38]. However, printed TPU conduits must be capable of withstanding the local mechanical stresses present in the tissue environment and of maintaining their structural integrity. The maximum strain achieved by the printed TPU (338 ± 3% for 90A and 370 ± 20 for 95A) specimens greatly exceeded the values reported for peripheral nerves (61± 2%–164 ± 34%) [38], indicating the capacity to undergo deformation before failure. In addition, successful nerve regeneration has previously been achieved using TPU 95A conduits, which may be attributed, at least in part, to the favorable mechanical properties of the polymer, providing a suitable mechanical environment for nerve regeneration [39]. Data normality was evaluated using the Shapiro–Wilk test, and all datasets followed a normal distribution. Therefore, comparisons of the mechanical properties between TPU 95A and TPU 90A were performed using Welch’s *t*-test. Differences in the mean values of elastic modulus, maximum stress, and strain between the TPU filaments were considered statistically significant at *p* < 0.05. All analyses were performed using OriginPro 2018 software.

### 3.5. 3D Printing of Nerve Conduits

**Figure 5:**
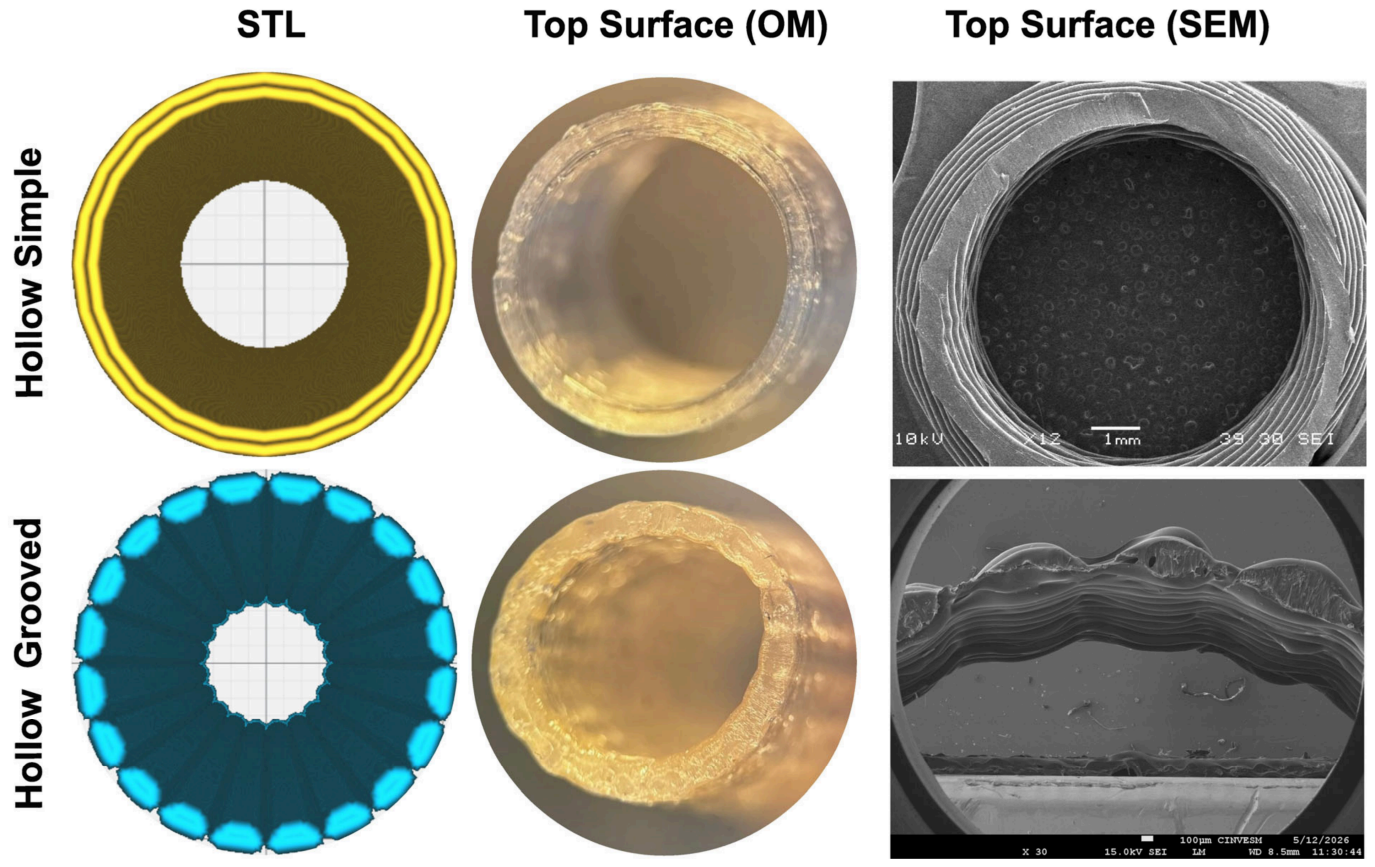
STL model, representative optical appearance, and SEM morphology of the fabricated hollow simple wall and hollow grooved conduits for TPU 90A.

**Figure 6:**
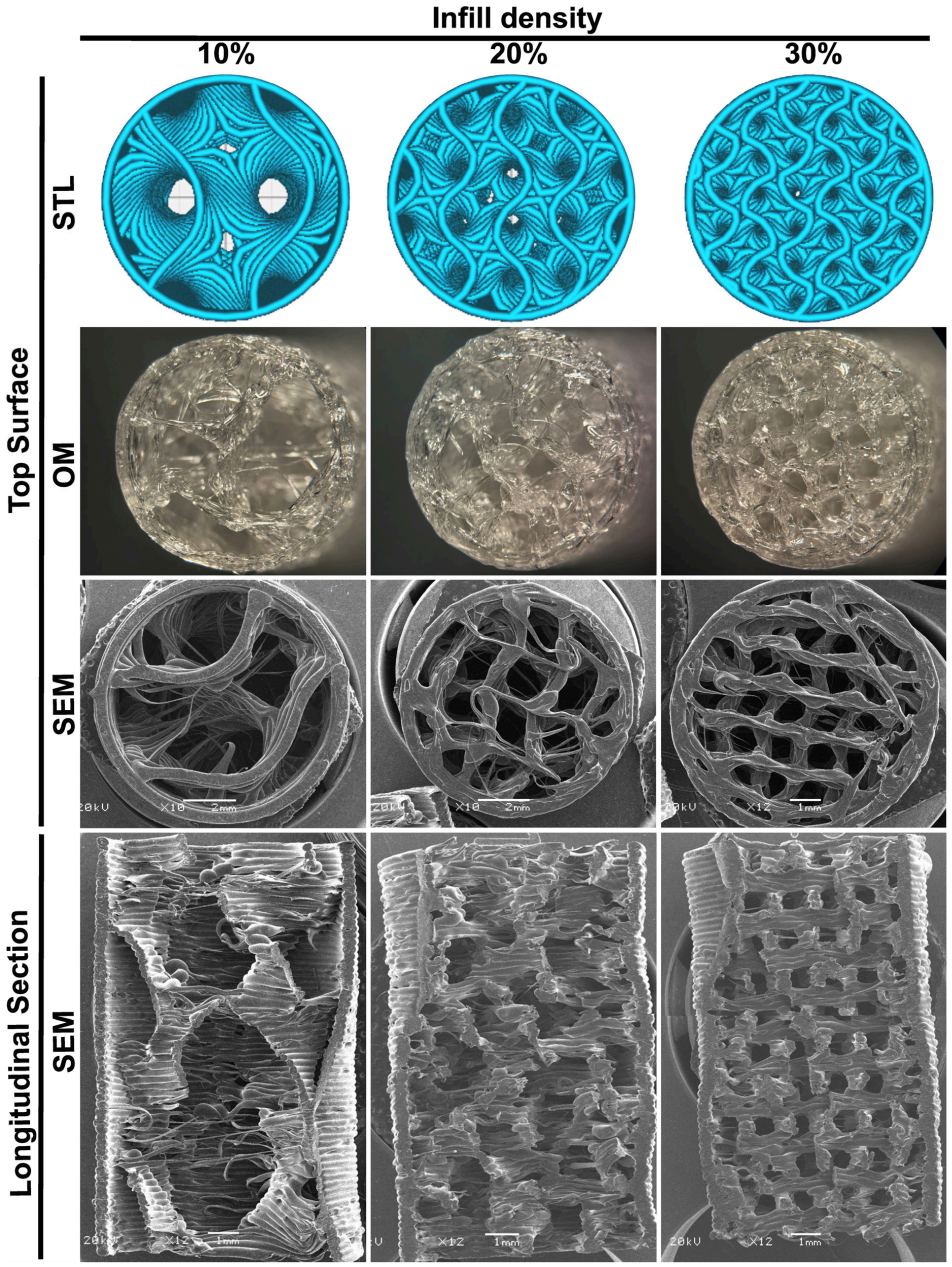
STL models, representative optical images (OM), and SEM micrographs of fabricated multichannel nerve guidance conduits with gyroid architectures and different infill densities (10%, 20% and 30%) for TPU 90A.

Representative scanning electron microscopy (SEM) (using a JEOL JSM-6360LV, 20 kV; Tokyo, Japan; gold sputter-coated samples) and optical microscopy (OM) images of conduits fabricated from TPU 90A are shown in Figure 5 and Figure 6. Similar architectures were obtained for conduits 3D printed from TPU 95A. Both the single hollow and grooved designs were successfully manufactured with high dimensional fidelity, indicating that the selected printing parameters enable accurate reproduction of the STL geometries.

On the other hand, SEM observations of the longitudinal sections of multichannel conduits revealed a homogenous gyroid-based interconnected architecture regardless of the infill density employed (see Figure 6). Although the gyroid pattern does not generate straight channels, it provides a continuous and highly interconnected three-dimensional network, which may facilitate nutrient diffusion and tissue infiltration. Furthermore, the consistent architecture observed across all infill densities demonstrates the reproducibility of the printing process. Indeed, there is evidence supporting the advantages of multichannel conduits over single hollow structures, as they promote enhanced remyelination and a higher density of Schwann cells during nerve regeneration [40,41]. Previous studies have frequently employed circular and honeycomb aligned microchannels in the design of NGCs [40,42,43]. To the best of our knowledge, the use of a gyroid-based internal structure has been scarcely explored.

Interestingly, studies employing anatomically inspired conduits derived for micro-computed tomography reconstruction have shown that the nerve fascicles do not necessarily follow perfectly straight and parallel pathways [44]. Instead, they often exhibited complex trajectories with gradual changes in orientation. Although patient-specific, anatomically inspired conduits may offer advantages, their fabrication is not always feasible. In such cases, a standardized and reproducible internal architecture could provide a beneficial structural feature for nerve regeneration. Based on these observations, the gyroid pattern employed in this study provides the NGC with a highly curved and interconnected network, potentially better resembling the native fascicular organization of peripheral nerves. This architecture could also provide continuous pathways that may contribute to axonal guidance, although this effect requires further experimental validation. Furthermore, computational fluid dynamics studies have demonstrated that gyroid scaffold networks increase the internal surface area and promote efficient fluid transport while providing a uniform distribution of wall shear stress [45]. Therefore, conduits with this multichannel architecture could enhance the viability and adhesion of Schwann cells and facilitate the transport of nutrients, oxygen and metabolic waste during nerve regeneration.

Additionally, previous studies have demonstrated that 3D-printed TPU 95A nerve guidance conduits support neuronal cell adhesion, proliferation, and gene expression [44]. Therefore, the combination of TPU and 3D printing, together with the incorporation of complex internal channel structures, suggests that fabricated multichannel NGCs may represent a promising platform for peripheral nerve regeneration.

### 3.6. Compression Test of 3D-Printed Nerve Conduits

Table 3 depicts the compressive mechanical properties of 3D-printed nerve conduits with TPU 90A and TPU 95A. The results of the radial compression test are expressed in terms of the load at 50% of diametral deformation, as well as the compressive modulus $\left(E_{s}\right)$, which was used as a structural parameter in lateral compression (Table 3) (the load values at 10%, 20%, 30% and 40% strain are described in Table S1, Supplementary Material).

**Figure 7:**
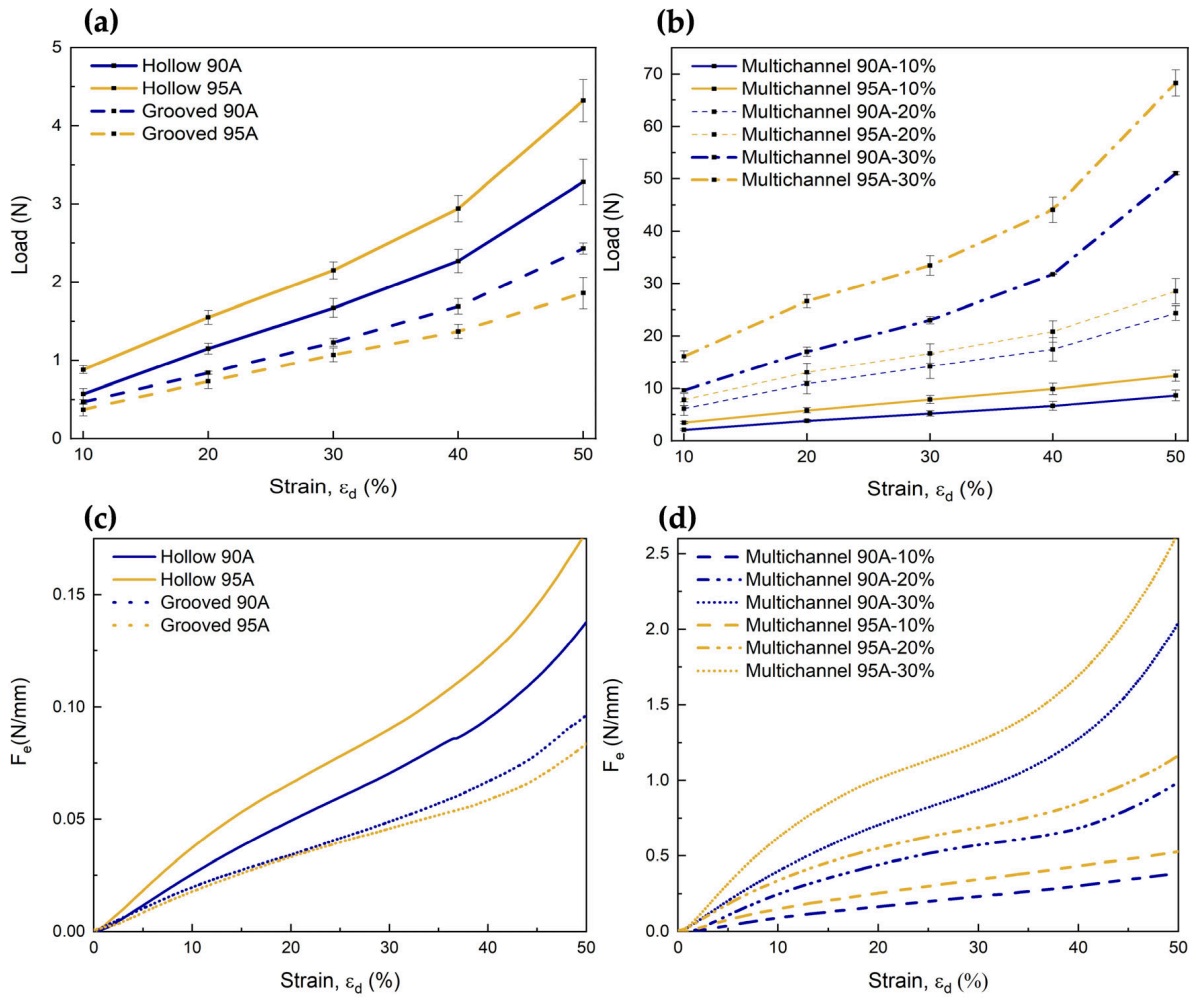
Compression test. Load recorded at 10, 20, 30, 40, and 50% diametral strain of (**a**) hollow, grooved (**b**) and multichannel conduits. Illustrative F_e_ vs. strain curves of (**c**) hollow, grooved and (**d**) multichannel conduits.

The compressive modulus of the hollow conduits increased from 0.28 ± 0.02 to 0.37 ± 0.04 N/mm when the TPU filament was changed from 90A to 95A. In contrast, grooved conduits exhibited a slight decrease in compressive modulus when the stiffness of the TPU filament was increased, from 0.185 ± 0.016 N/mm for TPU 90A to 0.16 ± 0.03 N/mm for TPU 95A. Hollow conduits withstood higher loads at every level of diametral deformation evaluated (10–50%) (See Figure 7a,c), owing to their denser walls and greater material content, whereas grooved conduits contain sections with less material, resulting in lower load-bearing capacity.

In addition, hollow NGCs fabricated from TPU 95A exhibited a higher load-bearing capacity and compressive modulus than those fabricated from TPU 90A, which is attributable to the greater stiffness of the 95A material due to its higher content of rigid segments. Unexpectedly, grooved conduits fabricated from the 90A filament exhibited slightly higher values of load and $E_{s}$ than those fabricated from 95A filament with the same morphology. Although the difference was small, these results suggest that the mechanical response of grooved morphology is influenced not only by intrinsic material properties such as stiffness but also by a structural factor (porosity, trapped air bubbles, adhesion between layers) and the deformation mechanism induced by the grooves.

When multichannel conduits were tested, the compressive modulus increased with TPU hardness (See Figure 7c,d) as in the case of hollow NGCs. Specifically, the modulus increased from 0.97 ± 0.08 for 90A TPU to 1.64 ± 0.16 N/mm for 95A TPU at 10% infill, from 2.7 ± 0.6 to 3.60 ± 0.06 N/mm at 20% infill, and from 3.89 ± 0.02 to 7.8 ± 0.5 N/mm at 30% infill. In addition, the multichannel conduits exhibited a gradual increase in compressive modulus with higher infill density, reaching values of 0.97 ± 0.08, 2.7 ± 0.5, and 3.89 ± 0.02 N/mm at 10, 20, and 30%, respectively, for TPU 90A and 1.64 ± 0.16, 3.60 ± 0.06, and 7.8 ± 0.5 N/mm at 10, 20, and 30%, respectively, for TPU 95A. In general, variations in infill density produced a more pronounced enhancement in the compressive properties than changes in TPU hardness. As the infill density increases, the internal structure of the conduits becomes less porous, providing a greater amount of material available to support the applied load and increasing the number of load-bearing pathways throughout the gyroid pattern [46].

Whereas the higher hard segment content of TPU 95A (54.1%) provides higher mechanical properties to improve the ability of the conduit to maintain its lumen, the lower hard segment content of TPU 90A (45.5%) provides greater flexibility, which may allow better adaptation of Schwann cells to the mechanical environment without compromising the stability of the conduits. As its elastic modulus is closer to that of native nerve tissues, it could render an appropriate balance during nerve regeneration. In addition, the slower degradation of TPU 95A may help maintain conduit integrity for longer periods, whereas the faster degradation of TPU 90A may be suitable for applications requiring earlier scaffold degradation. Thus, hard segment content is a key parameter for balancing mechanical stability, deformability, and degradation behavior.

Radial compression studies reported in the literature include conduits fabricated from different materials and exhibiting a wide range of dimensions and internal architectures. Nevertheless, such comparisons remain useful for determining whether the compression forces obtained in the present study fall within the range reported for other nerve guidance conduits. In general, compressive moduli of the 3D-printed conduits were comparable to or higher than values reported for other NGC, including PLGA-based conduits (0.37–1.44 N/mm), collagen conduits (0.3–1.8 N/mm), and commercial devices such as NeuraGen (1.8 N/mm) and Neurotube (3.7 N/mm) [47,48].

### 3.7. Cell Viability

**Figure 8:**
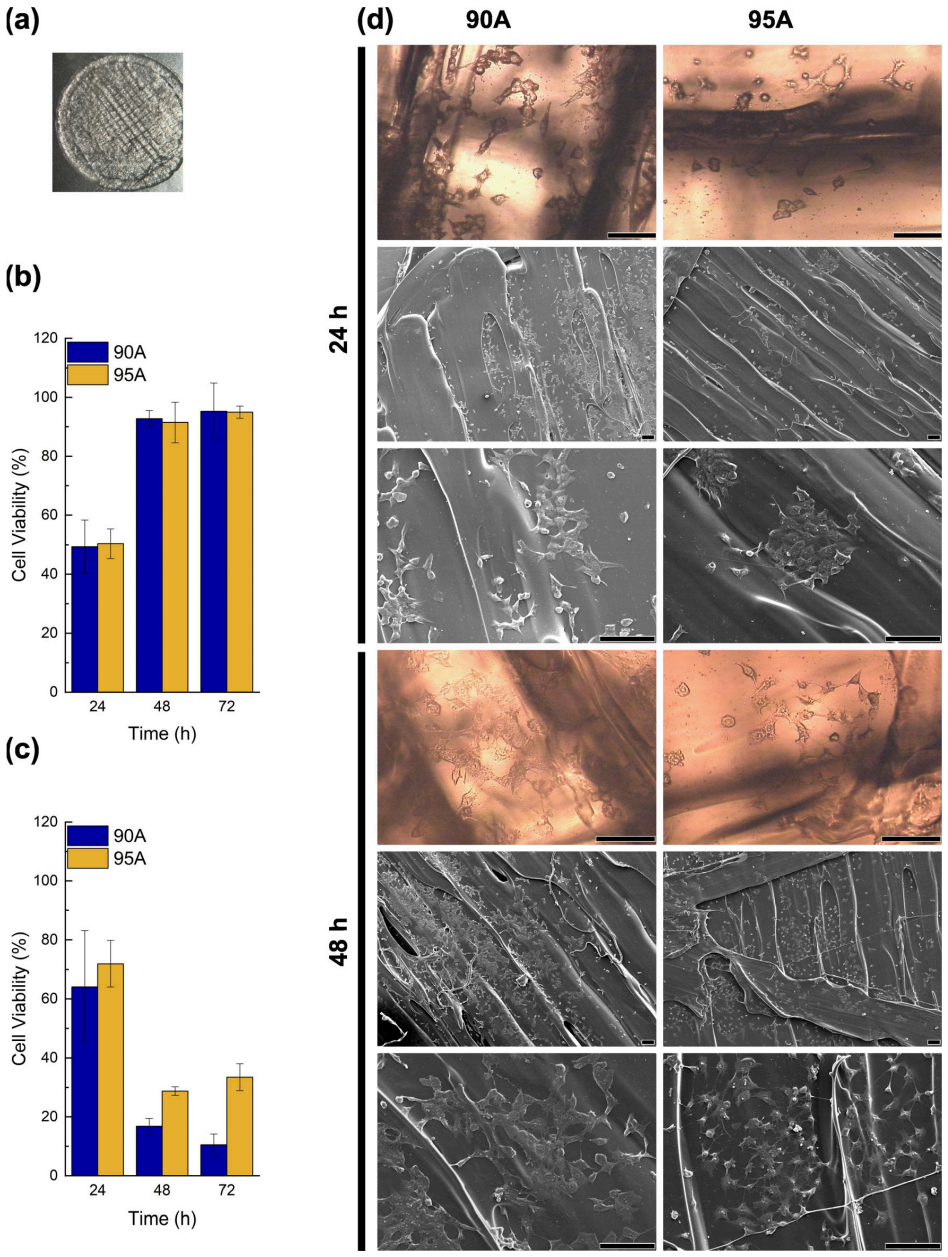
(**a**) 3D-printed TPU disc used in indirect and direct cell viability assays. Cell viability of fibroblasts: (**b**) indirect assay (extracts), (**c**) direct contact assay, (**d**) OM and SEM micrographs of fibroblasts cultured for 24 h and 48 h. Scale bars: 1 μm.

Figure 8b shows the viability of cells exposed to extracts obtained from 3D-printed TPU 90A and TPU 95A discs (Figure 8a). After 24 h of exposure, cell viability was approximately 50% (cytotoxic). However, after 48 and 72 h, cell viability increased to approximately 90% and remained relatively constant over time, indicating favorable cell proliferation and survival in the presence of the extracts. Statistical analysis using Student’s *t*-test revealed no significant differences in cell viability between the two materials. This result is likely related to the similar chemical composition of both thermoplastic polyurethanes, PBA, MDI and BO. In addition, the results are consistent with the accelerated degradation studies in water, which showed minimal degradation of the materials. Therefore, it is likely that the TPU extracts did not undergo significant changes during the cytotoxicity assay and, consequently, did not release degradation products at concentrations capable of inducing cytotoxic effects.

In contrast, the direct contact assay with fibroblasts cultured on TPU printed discs yielded low viability values (see Figure 8c). Cell viability was approximately 60% after 24 h and decreased around 20% after 48 h. However, OM and SEM (using an FESEM-7600 JEOL, Tokyo, Japan; gold and palladium sputter-coated samples) micrographs revealed many fibroblasts attached to the TPU surfaces (after 24 and 48 h) exhibiting an elongated, spindle-shaped morphology and forming an interconnected cellular network through extensive cell–cell contacts (see Figure 8d).

Although the resazurin assay indicated a reduction in cellular metabolic activity, microscopic analysis revealed extensive cell attachment and spreading on the polyurethane surface. The presence of well-developed cellular extensions suggests favorable cell–material interactions. These findings indicate that reduced metabolic activity does not necessarily imply cytotoxicity and should be interpreted together with cell morphology when evaluating the biological performance of polyurethane-based biomaterials.

On the other hand, the difference in cell viability between the indirect (extract) and direct contact assays is expected because they evaluate different aspects of the biological response. While the extract assay primarily assesses the effects of compounds released from the material, the direct contact assay exposes cells to the material surface, where the cellular response may also be influenced by surface-related characteristics, such as roughness and features inherent to the FDM manufacturing process. Consequently, the measured metabolic activity may differ between both assays. Therefore, the biological performance of the TPU should be interpreted based on the combined evaluation of metabolic activity and cell morphology rather than on either assay alone.

**Figure 9:**
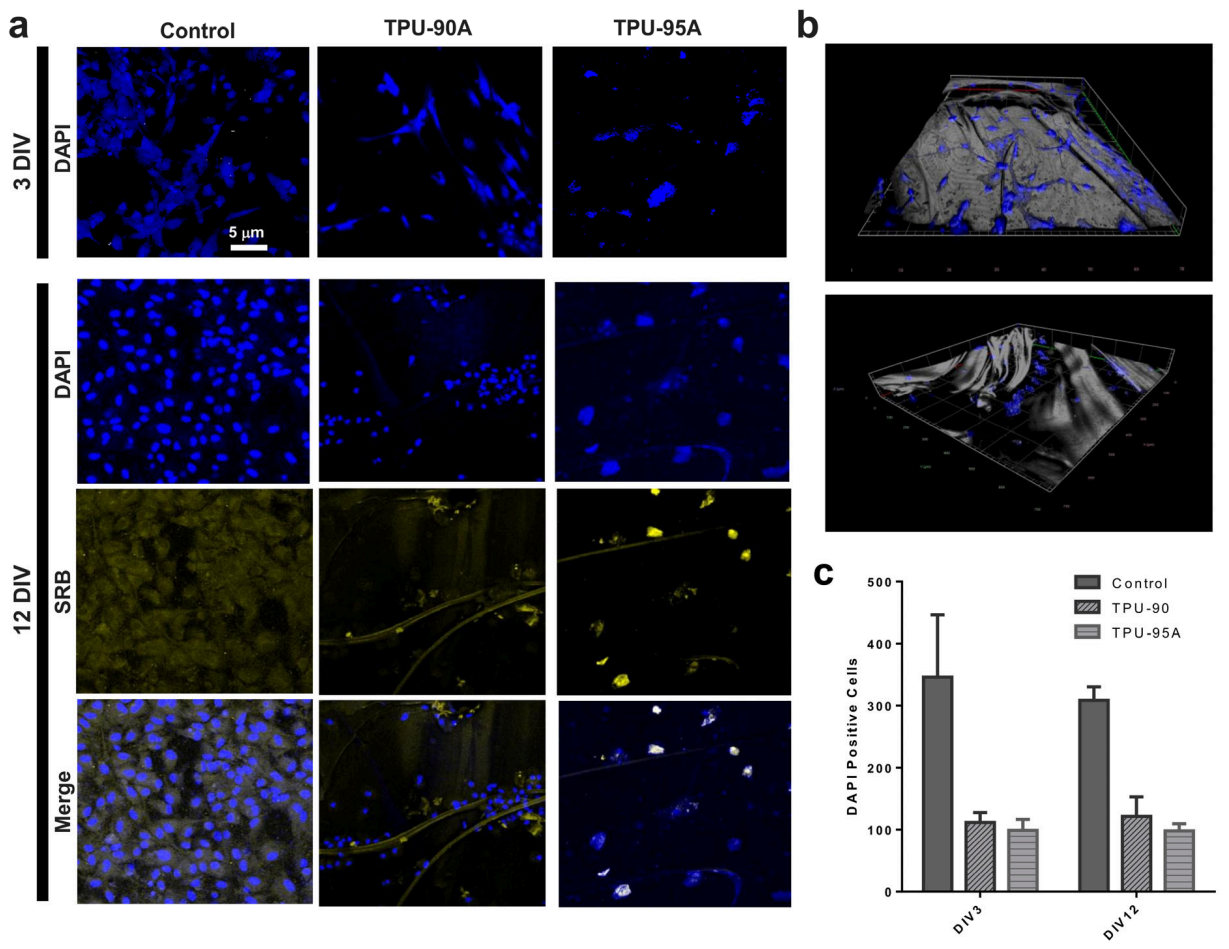
Biocompatibility and functional assessment of primary cortical mixed glial cell cultures grown on polymeric substrates. (**a**) Representative fluorescence micrographs of primary cortical mixed glial cell cultures grown on control glass coverslips and on the polymeric substrates TPU-90 and TPU-95A at 3 and 12 days in vitro (DIV). Nuclei were stained with DAPI (blue), and cytoplasmic morphology was visualized with Sulforhodamine B (SRB, yellow). (**b**) Three-dimensional reconstructions of confocal fluorescence and confocal reflectance images. (**c**) Quantification of DAPI-positive nuclei at 3 and 12 DIV.

The previous screening cytotoxicity test with fibroblast may suggest poor biocompatibility, but cells respond in a different manner depending on various factors. For the intended application as NGCs, primary cortical mixed glial cells were used. Fluorescence microscopy (Figure 9a) revealed that primary cortical mixed cultures adhered and survived on all tested substrates. At both 3 and 12 days in vitro (DIV), DAPI staining showed well-defined nuclei with no evidence of fragmentation or condensation, indicating preserved nuclear integrity across all conditions. Sulforhodamine B staining further confirmed intact cytoplasmic morphology, with cells displaying normal soma size and distribution regardless of the underlying material. Three-dimensional reconstructions (Figure 9b) of the cultures grown on the polymeric substrates demonstrated a continuous and homogeneous spatial distribution of cells, without signs of detachment, clustering, or irregular adhesion patterns. These observations indicate that both materials support stable cell–substrate interactions.

Quantification of DAPI-positive nuclei (Figure 9c) showed comparable cell densities across materials at both time points. Although the control condition exhibited higher cell adhesion, the TPUs also supported cell attachment, indicating that cells were able to adhere to their surfaces. Interestingly, the number of DAPI-positive nuclei remained relatively constant between DIV 3 and DIV 12 for control and printed TPUs.

The results demonstrate that the polymeric materials evaluated in this study are biocompatible and capable of supporting the survival, morphology, and functional responsiveness of primary cortical mixed cultures. The preservation of nuclear and cytoplasmic integrity, together with stable cell densities over time, indicates that none of the materials exert cytotoxic or inhibitory effects on cell viability. The uniform distribution observed in the 3D reconstructions further suggests that the physicochemical properties of these substrates are suitable for neural cell adhesion and maintenance. Overall, the findings support the suitability of the TPU materials for applications involving neural cultures, including biomaterial testing, neurophysiological assays, and the development of bioactive interfaces. Considering the favorable interactions observed with primary neural cells, it is expected that TPUs may also be compatible with other cell types including Schwann cells, which are key mediators of peripheral nerve regeneration. Although this study does not directly assess axonal regeneration or myelination, the sustained viability and preserved morphology of glial populations cells which are involved in metabolic support and myelin-related functions suggested that TPU substrates provided a permissive environment for relevant neural cell types.

Overall, a slight increase in fibroblast viability was observed for TPU 95A, which has a higher hard segment content, up to 48 and 72 h. However, no significant differences were observed in glial cell viability after 12 DIV.

## 4. Conclusions

The characterization performed in this study demonstrated that commercial TPU 90A and 95A based on PBA-MDI-BO possess a balanced combination of chemical, mechanical and structural properties suitable for the development of nerve guidance conduits fabricated using 3D printing by FDM. The combined evaluation of metabolic activity and cell morphology of fibroblasts indicated that the biological response should be interpreted using complementary tests. Furthermore, the favorable interactions observed with primary neural cells suggest potential compatibility with cells involved in peripheral nerve regeneration. From the different possible designs studied, multichannel conduits with gyroid infill architectures and higher rigid segment content showed enhanced resistance to radial compression while providing a biomimetic internal structure. These results are preliminary evidence of biocompatibility; thus, evaluating TPU-based conduits’ applicability in peripheral nerve regeneration will require further extensive and basic studies, involving, for example, Schwann cell performance, axonal growth ability, and models with increasing cellular complexity, among many others.

## Figures and Tables

**Table 1:** Principal 3D-printing parameters of human NGC.

Printing Parameters	Hollow	Grooved	Multichannel
Nozzle diameter (mm)	0.4	0.4	0.4
Wall thickness (mm)	0.5	0.3	0.25
Wall line count	1	1	1
Layer height (mm)	0.2	0.2	0.2
Extrusion temperature (°C)	225	225	225
Bed temperature (°C)	35	35	35
Printing velocity ($mm\cdot s^{-1}$)	30	40	30
Material flow (%)	100	100	100
Cooling (%)	100	100	100
Infill (%)	0	0	10, 20, 30
Mode	Spiralized	Layer by layer	Layer by layer

**Table 2:** Uniaxial tension test. Mechanical properties of 3D-printed specimens.

3D-Printed TPU	HS%	E (MPa)	$\sigma_{\max}$(MPa)	$\epsilon_{\max}$(%)
90A	45.5	20.8 ± 1.4	10.1 ± 0.1	338 ± 3
95A	54.1	30.6 ± 1.7	22.7 ± 1.5	370 ± 20

**Table 3:** Compression test of NGC.

3D-Printed NGC *	Load (N) **	$E_{s}$ (N/mm)
Hollow 90A	3.3 ± 0.3	0.28 ± 0.02
Hollow 95A	4.3 ± 0.3	0.37 ± 0.04
Grooved 90A	2.43 ± 0.07	0.19 ± 0.07
Grooved 95A	1.9 ± 0.2	0.16 ± 0.03
Multichannel 90A-10%	8.6 ± 1.0	0.97 ± 0.08
Multichannel 95A-10%	12.4 ± 1.1	1.64 ± 0.16
Multichannel 90A-20%	24.4 ± 1.4	2.7 ± 0.6
Multichannel 95A-20%	28 ± 2	3.60 ± 0.06
Multichannel 90A-30%	51.0 ± 0.3	3.89 ± 0.02
Multichannel 95A-30%	68 ± 2	7.8 ± 0.5

* NGC type (hollow, grooved, or multichannel)—TPU filament hardness (90A or 95A)—infill percentage (10%, 20%, or 30%; multichannel conduits only). ** Load recorded at 50% diametral deformation in compression.

## Data Availability

The data presented in this study are available upon request from the corresponding author.

## Supplementary Materials

The following supporting information can be downloaded at: https://www.mdpi.com/article/10.3390/polym18151865/s1, Figure S1: TGA and DSC of PBA; Table S1: Conduits dimensions; Table S2: Load at 10, 20, 30, 40% strain.
