# Supplementary material for: Evaluation of 3D-Printed Thermoplastic Polyurethane Nerve Guidance Conduits
Source: Polymers (Basel). 2026 Jul 29;18(15):1865. doi: 10.3390/polym18151865 (PMC13467680; doi:10.3390/polym18151865)
Supplement: Supplementary file 1 [file polymers-18-01865-s001.zip › polymers-4417515-supplementary.pdf]

Evaluation of 3D-Printed Thermoplastic Polyurethane Nerve Guidance Conduits

Alexis B. Sabido-Barahona [1], Rossana F. Vargas-Coronado [1], Claudia Vásquez-López [2], Abraham J. Cisneros-Mejorado [3], Rainald Pablo Ordaz [3], Reinher Pimentel-Domínguez [4], Antònia Colom-Casasnovas [3], Rogelio O. Arellano [3], Juan V. Cauich-Rodríguez [1,*] and Angel Marcos-Fernández [5,*]

1 Materials Department, Centro de Investigación Científica de Yucatán, A. C., Calle 43 No. 130 x 32 y 34, Col. Chuburná de Hidalgo, Mérida 97205, YUC, Mexico; alexis.sabido@estudiantes.cicy.mx (A.B.S.-B.); ross@cicy.mx (R.F.V.-C.)
2 Departamento de Física, Universidad de Sonora, Encinas y Rosales, s/n., Hermosillo 83000, SON, Mexico; claudia.vasquez@iq.uson.mx
3 Laboratorio de Neurofisiología Celular, Instituto de Neurobiología, Universidad Nacional Autónoma de México (UNAM), Boulevard Juriquilla 3001, Querétaro 76230, QRO, Mexico; acisneros@secihti.mx (A.J.C.-M.); pablo.ordaz88@comunidad.unam.mx (R.P.O.); a.colomcasasnovas@gmail.com (A.C.-C.); arellano.ostoa@comunidad.unam.mx (R.O.A.)
4 Centro de Física Aplicada y Tecnología Avanzada, Universidad Nacional Autónoma de México (UNAM), Boulevard Juriquilla 3001, Querétaro 76230, QRO, Mexico; reinherp@fata.unam.mx
5 Institute of Polymer Science and Technology (ICTP-CSIC), Juan de la Cierva, 3, 28006 Madrid, Spain
* Correspondence: jvcr@cicy.mx (J.V.C.-R.); amarcos@ictp.csic.es (A.M.-F.).

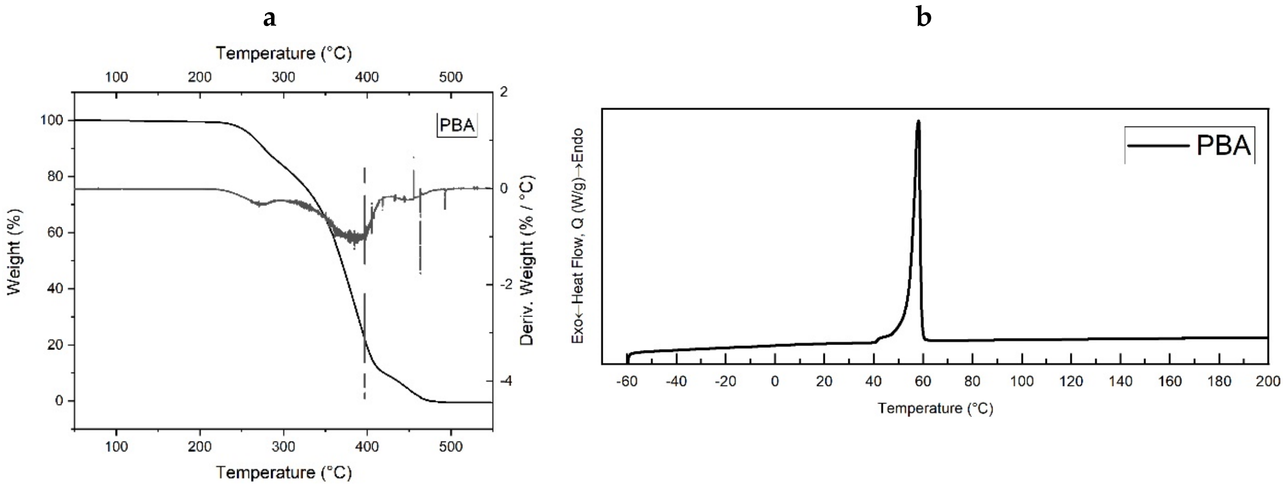

Figure S1. (**a**) TGA and (**b**) DSC of PBA.

Table S1. Conduits dimensions.

**3D- printed NGC**	d_e (mm)	WT (mm)	CZ (mm)
Hollow 90A	8.12 ± 0.12	0.41 ± 0.08	-
Hollow 95A	8.18 ± 0.32	0.43 ± 0.12	-
Grooved 90A	8.12 ± 0.08	-	-
Grooved 95A	8.19 ± 0.16	-	-
Multichannel 90A-10%	8.41 ± 0.16	0.50 ± 0.02	3.63 ± 0.20
Multichannel 95A-10%	8.45 ± 0.21	0.50 ± 0.04	3.65 ± 0.23
Multichannel 90A-20%	8.33 ± 0.08	0.52 ± 0.03	1.12 ± 0.12
Multichannel 95A-20%	8.35 ± 0.05	0.51 ± 0.05	1.15 ± 0.17
Multichannel 90A-30%	8.27 ± 0.12	0.43 ± 0.04	0.65 ± 0.02
Multichannel 95A-30%	8.22 ± 0.15	0.45 ± 0.02	0.66 ± 0.07

Outer diameter (d_e): 8 mm, wall thickness (WT) and Channel Size (CZ). The grooved conduits exhibited different wall thicknesses as a consequence of their morphology; however, a detailed morphological characterization was not the primary focus of this study.

Table S2. Load at 10, 20, 30, 40% strain.

**3D- printed NGC**	Load at 10% (N)	Load at 20% (N)	30% Load at 30%	Load at 40%
Hollow 90A	0.57 ± 0.07	1.15 ± 0.07	1.67 ± 0.12	2.27 ± 0.15
Hollow 95A	0.88 ± 0.05	1.55 ± 0.09	2.15 ± 0.11	2.94 ± 0.17
Grooved 90A	0.47 ± 0.03	0.84 ± 0.02	1.23 ± 0.05	1.69 ± 0.1
Grooved 95A	0.37 ± 0.08	0.73 ± 0.09	1.07 ± 0.09	1.86 ± 0.2
Multichannel 90A-10%	2.11 ± 0.16	3.82 ± 0.27	5.23 ± 0.52	6.67 ±0.82
Multichannel 95A-10%	3.49 ± 0.23	5.82 ± 0.5	7.89 ± 0.77	9.88 ± 1.06
Multichannel 90A-20%	6.1 ± 1.27	10.9 ± 1.91	14.2 ± 2.34	17.4 ± 2.23
Multichannel 95A-20%	7.9 ± 1.2	13.0 ± 1.7	16.7 ± 1.9	20.9 ±2.0
Multichannel 90A-30%	9.63 ± 0.3	17.02 ± 0.9	22.97 ± 0.7	31.82 ± 0.1
Multichannel 95A-30%	16.1 ±1	26.6 ±1.2	33.5 ±1.9	44.1 ±2.4
